# Systematic evaluation of bulk RNA deconvolution techniques identifies cell type compositional changes in Alzheimer's Disease brains

**DOI:** 10.1002/alz70855_105311

**Published:** 2025-12-24

**Authors:** Jaclyn Beck, Vilas Menon

**Affiliations:** ^1^ Sage Bionetworks, Seattle, WA, USA; ^2^ Columbia University Irving Medical Center and the Taub Institute for Research on Alzheimer's Disease and the Aging Brain, New York, NY, USA

## Abstract

**Background:**

Alzheimer's disease has differing effects on brain regions and cell populations as it progresses. Examining vulnerable cell populations can be difficult at high resolution, however, because large‐scale single cell transcriptomics is expensive, and differential survivability of cells can affect population distribution and quality. Bulk RNA sequencing allows for larger sample sizes and includes nuclear, cytoplasmic, and distal RNA, but does not provide cell‐specific data. In this study, we leveraged the benefits of both modalities by using existing single cell RNA data to deconvolve cell type proportions in bulk RNA samples of human brains from three large‐scale AD studies. We used the best outputs from multiple deconvolution strategies to estimate cell population changes associated with Alzheimer's disease.

**Method:**

We analyzed three bulk RNA data sets from donors with and without AD, provided by the Mayo RNAseq Study (*n* =  481), the Mount Sinai Brain Bank Study (*n* =  974), and the Religious Orders Study/Memory and Aging Project (ROSMAP) (*n* =  1598). Gene marker lists for each cell type were created using five AD‐related single‐cell RNA data sets (Cain 2020, Gabitto 2024, Lau 2020, Leng 2021, and Mathys 2019). We used six deconvolution algorithms (CIBERSORTx, DeconRNASeq, Dtangle, DWLS, MuSiC, and Scaden) with varying marker sets to estimate the cell type proportions in each bulk sample. We also tested different normalization and batch correction strategies. The three best‐fitting parameter sets from each algorithm were used to calculate cell type proportion differences between AD and control samples.

**Result:**

We found significant AD‐related decreases (*p* < 0.01) in 5 of the 17 neuronal subtypes we analyzed, replicated across multiple algorithms and brain tissues. We also found significant increases in astrocytes and oligodendrocytes. Methodologically, we found that CIBERSORTx, dtangle, and MuSiC, combined with markers generated from the Mathys 2019 data, produced the best results.

**Conclusion:**

Our research identified five neuronal subtypes that may be more vulnerable to Alzheimer's disease. This knowledge can motivate more experiments at single‐cell resolution that focus on these populations. Our experimentation with different deconvolution methods also yielded useful information for future analysis on similar data with closely‐related or rare cell types.

